# The Dietary and Non-Dietary Management of Osteoporosis in Adult-Onset Celiac Disease: Current Status and Practical Guidance

**DOI:** 10.3390/nu14214554

**Published:** 2022-10-28

**Authors:** Abdulbaqi Al-Toma, Amin Herman, Willem F. Lems, Chris J. J. Mulder

**Affiliations:** 1 Department of Gastroenterology and Hepatology, St. Antonius Hospital, 3435 CM Nieuwegein, The Netherlands; 2 Department of Rheumatology, St. Antonius Hospital, 3435 CM Nieuwegein, The Netherlands; 3 Department of Rheumatology and Clinical Immunology, Amsterdam UMC, Location Vrije Universiteit, 1081 HV Amsterdam, The Netherlands; 4 Department of Gastroenterology, Amsterdam UMC, Location Vrije Universiteit, 1081 HV Amsterdam, The Netherlands

**Keywords:** celiac disease, osteoporosis, bone mineral density, prevention, malabsorption, gluten-free diet, fracture

## Abstract

Impaired bone mineral density (BMD) is a frequent complication of adult-onset celiac disease (CeD). This is usually due to malabsorption of nutrients, changes in bone metabolism in association with inflammation, and to a lesser extent, decreased overall physical health and mobility. This review aims to highlight the current status concerning surveillance, prevention, and treatment strategies for bone disease in CeD. A practical guidance on these matters is suggested. The available published research on the prevention and treatment of decreased BMD in relation to CeD is scarce. In general, publications were based on expert opinions or extrapolation from studies on postmenopausal women or inflammatory bowel disease. Optimal dietary treatment and an adequate supply of calcium and vitamin D are the cornerstones for the reduction in fracture risk in patients with CeD. In adults with low BMD or fragility fractures, CeD needs to be considered and specifically approached. When osteoporosis is documented, start treatment with an antiresorptive agent; these agents are proven to result in a long-term reduction in fracture risk in high-risk individuals. However, there are some important differences between the management of male and female patients, particularly premenopausal women, that need to be addressed. In patients with persisting diarrhea and malabsorption, parenteral medications may be preferable. Future research specifically focusing on celiac disease and the associated disorders in bone mineralization is mandatory to provide evidence-based recommendations in this field.

## 1. Introduction

### 1.1. Celiac Disease

Celiac disease (CeD) is a life-long gluten-related enteropathy triggered by gluten ingestion in susceptible subjects. [1,2]. Genetic background is an essential prerequisite for the development of the disease (HLA-DQ2/DQ8 positivity and non-human leukocyte antigen (HLA) genes), but the contribution of other non-genetic factors such as viral infections and gut dysbiosis might also be important [3,4]. It has been suggested that gut permeability and dysbiosis play a key pathogenic role in CeD through an impaired expression of zonulin.

CeD is considered a global burden since its prevalence reaches almost 1% worldwide, making it one of the most common autoimmune disorders [5]. CeD is diagnosed in females 2–3 times more often than in males. The diagnosis of CeD may be made at all ages, even in the elderly; more than 70% of cases are diagnosed above the age of 20 years [6].

Although CeD is readily treatable with a gluten-free diet (GFD), patients need a structured follow-up to detect and avert long-term complications and achieve a good quality of health.

Non-adherence to a GFD is a notable cause of continuing gut inflammation and a decrease in quality-of-life [7]. The spectrum of CeD symptomatology is broad, ranging from asymptomatic disease detected at screening to a clinical condition characterized by wasting, undernutrition and steatorrhea to several selective deficiencies of nutrients, potentially resulting in extra-gastrointestinal features such as impaired bone mineral density (BMD) and vertebral and non-vertebral fractures [5].

CeD has a benign course in the majority of patients; however, almost <0.5% of adult celiac patients are refractory to GFD. Severe malnutrition, nutritional deficiencies and impaired bone health are regularly found in this subgroup [8].

### 1.2. Osteoporosis in Celiac Disease

Osteoporosis is a skeletal disorder characterized by reduced BMD, deteriorated microarchitecture, and reduced strength of bone, with susceptibility to fragility or low-impact fractures [9,10]. Generally, osteoporosis remains an underdiagnosed and undertreated condition, which represents a substantial health care problem [11]. There are high annual direct costs of osteoporosis and the mortality following a hip fracture in the elderly is as high as 24% [12]. Pelvic and/or humeral low-energy fractures are frequent in association with osteoporosis and contribute to high rates of morbidity and mortality [13]. Psychosocial complaints, in particular depression, are usual consequences of fracture, because of pain, limitation of physical activities, and deprivation of independence. About, 60% of survivors of hip fractures do not attain a pre-fracture level of physical independence, and 20% of them will be nursing home residents. Therefore, timely diagnosis of osteoporosis and taking measures to prevent fractures are vital to decreasing mortality and preserving the independence of people at risk for fragility fracture [14].

Osteoporosis might be the sole presentation of undiagnosed CeD without gastrointestinal symptoms or even detected later in the course of the disease [15,16,17]. The prevalence of osteoporosis in adults diagnosed with celiac disease is highly variable, probably depending, among other factors, on the severity and the duration of the condition when diagnosed. Decreased BMD is reported in >50% of newly diagnosed adult celiac patients [16]. CeD is found to be associated with decreased BMD in children and adults and is a recognized risk factor for osteoporotic fractures in men aged ≥40 years [18]. A meta-analysis by Ganji et al. reported an osteoporosis prevalence of 14.4% in CeD and 39.6% in osteopenia [18].

Studies, on the other hand, have found an increased prevalence of CeD in people with low BMD [19,20]. An appropriate estimation of CeD prevalence is 2–3% in those individuals with low BMD, in comparison with about 1% in the general population.

Multiple skeletal sites are affected by low BMD in CeD, particularly the neck of the femur and the lumbar spine. The trabecular bones are usually the sites of bone deterioration as compared to the cortical bone, which is less metabolically active [21]. CeD women have lower BMD and abnormal bone microarchitecture in comparison with non-celiac premenopausal women of similar age, body mass index, ethnicity, and race [22].

Celiac disease-related osteoporosis is associated with being underweight, age over 45 years, and male gender (in those younger patients) [23]. It is also with more severe intestinal histopathological changes [24].

Low BMD in pediatric celiac patients responds to GFD [25]. The same might be seen in adults after adequate treatment with GFD [25,26].

The change in BMD seems to happen particularly in the first year after starting a GFD [27]. In a prospective study, Newnham et al. [27] showed that bone mass improved in celiac patients over the first year after starting GFD and the degree of improvement was related to the T-score measured at diagnosis. The change was seen in those patients having osteopenia or osteoporosis, but no change was witnessed in those with normal BMD. Positive BMD changes improved the classification in 14% of individuals, with a shift from osteoporosis to osteopenia and then to a BMD in the normal range. At the assessment five years later, the changes were still significant. Therefore, mucosal improvement and healing as a consequence of adherence to GFD are linked with continuing improvement of the reduced bone mass. However, there is an important variability in response between individuals, and in most cases, BMD does not achieve normality. This is not unexpected because about 97% of peak bone mass is gained in the first two decades of life [28,29].

Studies dealing with the assessment of fracture risk in CeD patients had conflicting outcomes, depending on the duration of follow-up, degree of dietary compliance, analysis of fracture history, and mucosal status. In patients with CeD, the fracture risk varied from 1.3 to 10-fold more than that in the general population [30,31,32,33,34].

Jafri et al. [17] studied long-term fracture risk in CeD and reported that CeD is associated with an increased fracture risk both before and after diagnosis. Before the diagnosis, the fracture rate is twice that of controls, and the rate is 2.5-fold greater after the date of diagnosis. This applies to both appendicular and axial fractures.

Ludvigsson et al. [35] concluded that CeD was associated with subsequent hip fracture (hazard ratio = 2.1; 95% confidence interval (CI) = 1.8–2.4) and fractures at all sites (hazard ratio = 1.4; 95% CI = 1.3–1.5). This increased risk remains even 20 years after the diagnosis of CeD.

One study reported a 0.38% prevalence of biopsy-proven CeD in patients with fractures; 0.19% of them had a new CeD diagnosis. This lies within the range of prevalence in the Western European population (0.33–1.5%) [36].

### 1.3. Physiology and Pathophysiology of Alterations of Bone Health in Celiac Disease

The pathophysiology of decreased bone mineral density in CeD is multifactorial, including local and systemic mechanisms [21]. These can be summarized as follows:

Mucosal villous atrophy in CeD causes decreased calcium absorption, resulting in hypocalcemia and consequently secondary hyperparathyroidism [37]. The latter stimulates osteoclast-mediated bone resorption; which may lead to osteopenia or osteoporosis. Parathyroid hormone (PTH) is essential for the maintenance of serum calcium levels within narrow limits by actions on the kidneys and bone, and also by effects on the gastrointestinal tract. PTH is released tonically in a pulsatile fashion by the parathyroid gland. One of the important mechanisms through which PTH regulates calcium homeostasis is related to its stimulatory role in bone remodeling. PTH stimulates both bone resorption and formation, with the final outcome depending on the dose and periodicity of the PTH signal. Continuous PTH release has catabolic effects on the skeleton; on the other hand, intermittent PTH doses have an anabolic effect [38]. Vertebral fractures are common in hyperparathyroidism, even at higher BMD than in patients with osteoporosis. This might be explained by microarchitectural changes caused by the parathyroid hormone, which cannot be detected by BMD measurement [39].

Magnesium deficiency, which may occur in gluten-sensitive enteropathy, is known to impair the secretion and action of parathormone, resulting in osteopenia and increased skeletal fragility [40].

Concomitant hypogonadism, low insulin growth factor-1, zinc deficiency, and malnutrition contribute to bone loss by increasing bone resorption.

Systemic, chronic, low-grade inflammation: In CeD, there is a low-grade systemic inflammatory response with hypersecretion of inflammatory cytokines. These cytokines increase bone resorption and promote bone loss [41,42]. Furthermore, hypovitaminosis D is found to often be associated with systemic low-grade inflammation [43].

Tissue transglutaminase, a key immunological component in CeD, might be an important factor in bone metabolism by regulating receptor activator of nuclear factor kappa B (RANKL) and the differentiation of osteoblasts [44]. More research work is needed to explore this hypothesis.

Vitamin D contributes in many ways to bone mineralization, predominantly by maintaining calcium and phosphate homeostasis. It regulates intestinal calcium intake through the vitamin D receptor.

Vitamin D is activated by the renal enzyme 1-alpha-hydroxylase upon stimulation by PTH. In CeD, this process will lead to an increase in intestinal absorption of calcium by an increase in vitamin D-dependent active calcium transport [45,46]. Paradoxically, high levels of 1,25-vitamin D may cause bone resorption. Vitamin D_3_ (cholecalciferol) is the principal vitamin D from dietary sources, present mainly in foods of animal origin. However, the majority of vitamin D_3_ (estimated at 80%) is from endogenous production by the action of ultraviolet light on the skin. Diet may contain 25-hydroxy cholecalciferol (25OHD_3_) and also small quantities of dihydroxy cholecalciferol (1,25(OH)_2_D_3_) [46].

At low dietary concentrations, vitamin D uptake is principally protein-mediated, but there is also passive absorption when vitamin D is given at pharmacological doses [47]. The absorption of vitamin D is reduced in the presence of villous atrophy, in part due to the malabsorption of fat. Furthermore, fatigue and decreased activity, coinciding with a diminished nutritional condition, may result in decreased sun exposure with consequent vitamin D deficiency [16].

Calcium homeostasis is controlled by hormones (parathyroid hormone PTH, 1,25-dihydroxyvitamin D, and calcitonin) and organs: the small bowel, which regulates absorption; bone, which serves as a calcium reservoir; and the kidneys. When blood calcium concentration decreases, there will be a rapid increase in PTH release that promotes bone turnover and cortical bone loss. Thus, calcium malabsorption in CeD plays a pivotal role in the induction of a series of events that lead to bone demineralization. Hyperparathyroidism is frequent and should be detected in newly diagnosed patients as it is responsible for the acceleration of bone turnover. Calcium malabsorption is a consequence of steatorrhea, deficiency of vitamin D and defective vitamin D-dependent calcium absorption [45].

Deficient BMD in CeD may occur independently of gastrointestinal symptoms [48,49]. Additionally, at diagnosis, the severity of the histopathological changes could predict the occurrence of low BMD, which carries a risk of developing osteoporosis if left unaddressed [24,50].

## 2. Aims and Methods

Physicians dealing with CeD, both in the first and second lines, are in need of clear strategies for the management of bone disorders in association with CeD. This review aims to highlight the current status concerning surveillance, prevention, and treatment strategies for bone disease in CeD. A suggested practical guidance on these matters is provided.

PubMed, Google Scholar, Web of Sciences, and the Cochrane Central databases were searched to find the relevant published articles until August 2022. The search strategy was based on medical subject headings (MeSH) as follows: (celiac OR coeliac OR “gluten-sensitive enteropathy” OR sprue) AND (bone mineral density OR densitometry OR metabolic bone disorder OR osteoporosis OR osteopenia OR fragility fractures) AND adult. Only English-language literature was reviewed.

The search identified fully published research articles, international guidelines and abstracts dealing with CeD, osteoporosis and BMD. The authors revised the full text of the selected literature and evaluated the quality of the data presented. An overview of the diagnostic approach, management issues such as lifestyle, dietary therapies and pharmacological agents for osteopenia and/or osteoporosis in adult CeD patients are provided. The majority of the studies dealing with bone mineral density in CeD were observational studies. Therefore, we have built our conclusions on the limited literature on CeD and bone density and on the extrapolation of available evidence on osteoporosis in inflammatory bowel disease and postmenopausal women.

## 3. Assessment

### 3.1. Assessment of Bone Condition in Celiac Disease

#### 3.1.1. Clinical Assessment

Adult patients with CeD need to be questioned to identify risk factors that predispose to a decrease in bone mineral density and if there is a history of low-energy fractures. A detailed assessment is necessary of personal and family history, fracture risk, and clinical examination. The patient’s dietary habits, alcohol intake, and smoking need to be highlighted.

Past medical history of diseases associated with osteoporosis and CeD should be taken. Examples of such conditions are diabetes mellitus type 1, thyroid disorders, inflammatory bowel diseases, etc. Furthermore, attention should be paid to the use of medications that may be the cause or have a contributory role in the development of osteoporosis and/or fractures [51,52].

An assessment of fall risk is needed because a lot of fractures in the elderly are related to falls.

#### 3.1.2. Laboratory Tests

Serum calcium, albumin, alkaline phosphatase, and 25(OH) vitamin D concentrations in adults should be measured at the time of diagnosis and again during follow-up. In those patients who have a low BMD, renal function, parathormone, serum magnesium, and urinary calcium are needed. Serum levels of estrogen and testosterone may also be indicated [51,52].

#### 3.1.3. Dual-Energy X-ray Absorptiometry (DXA)

DXA measurement is the preferable radiological technique for confirming or excluding a diagnosis of osteoporosis, provides a prediction of fracture risk, and is also used in patient monitoring.

DXA is measured at the femoral neck (and/or total hip) and lumbar spine [53]. Osteoporosis is present when BMD is ≥2.5 standard deviations (SD) below the mean of a healthy reference population, and osteopenia is present when BMD is between −1 SD and −2.5 SD. Severe osteoporosis is regarded to be present when BMD is > 2.5 SD below the mean of a healthy reference population and there is a current or past history of fragility fracture [53].

#### 3.1.4. Vertebral Fracture Assessment

Together with DXA, it is recommended to perform a vertebral fracture assessment [54]. In an adult patient 50 years or older, the presence of a vertebral fracture is diagnostic of osteoporosis, even when DXA diagnosis is lacking. The presence of a single vertebral fracture indicates an increased risk of further vertebral fractures and/or fractures at other sites, such as the hip or other peripheral sites [55]. Unfortunately, the majority of vertebral fractures are clinically silent and asymptomatic. Therefore, they may stay undiscovered for many years. Notably, a high percentage of females with asymptomatic vertebral fractures have BMD levels that would not mandate pharmacological treatment on the basis of DMD results alone [56]. The detection of an earlier unrecognized vertebral fracture may alter the diagnostic classification, change the calculation of fracture risk, and dictate treatment decisions [57].

Pre-DXA fracture-risk status has a sensitivity of >80% in correctly recognizing celiac disease patients with osteoporosis who need to be treated [58].

#### 3.1.5. Fracture Risk Assessment (FRAX) Tool

FRAX is a tool used to make an estimation of an individual‘s 10-year fracture risk [59]. This tool may be used to select patients who should have a DXA scan and can help to determine a suitable therapeutic agent. The FRAX score is determined by ten factors: age (>40 years), gender (M/F), body mass index (BMI), fracture history (yes/no), presence of hip fracture in parents (yes/no), current smoking (yes/no), steroids use (yes/no), rheumatoid arthritis history (yes/no), secondary cause of osteoporosis (yes/no), and excessive alcohol (yes/no). One literature report showed that, because of FRAX’s high negative predictive value, this tool may be effective to avoid performing unnecessary DXA scans in patients with CeD [60].

#### 3.1.6. Timing of DXA Scan in Adult Celiac Disease

A baseline DXA scan is needed in adult-onset CeD. However, in the published international guidelines, there is no consensus as to when this measurement should be performed [5,61,62].

The following individualized approach is suggested:

i. DXA may be considered in establishing the diagnosis of CeD, particularly in the presence of
  - Malabsorption with significant weight loss or low body weight;
  - Delayed diagnosis (above 40 years) or in patients with severe CeD presenta-tion;
  - The presence of a history of fragility fracture or when an individual has an-other important risk factor for osteoporosis (such as rheumatoid arthritis, hyperparathyroidism, hypogonadism, thyroid gland disorders, hip fracture in the family, smoking and excess alcohol use);
  - Down syndrome: These patients have an estimated six-fold increased chance of having CeD in comparison to the general population [44]. An increased prevalence of osteoporosis in Down syndrome has been reported [45]. This is possibly attributed to the diminished osteoblastic bone formation with no significant differences in bone resorption.

ii. DXA in CeD without additional risk factors
  - A DXA scan may be performed later in the course of the disease, for example, at 30–35 years of age [4]. Pantaleoni et al. [33] reported that stratifying patients according to gender and age showed a higher prevalence of low BMD in men older than 30 years and in women of all ages. Therefore, they proposed that DXA needs to be performed when CeD is diagnosed in those older than 30 years of age.

iii. Follow-up DXA
  - When the initial DXA is normal, then it needs to be repeated every five years. In patients with osteopenia/osteoporosis or evidence of ongoing villous atrophy, 2–3-yearly DXA is required [4,24].

### 3.2. Celiac Disease Screening in Individuals having Fragility Fracture or Low BMD

When there are gastrointestinal symptoms or extraintestinal disorders known to be associated with CED, or when there is a positive family history for CED, the clinical awareness of an underlying CED increases. However, clinicians should be aware of CeD without classical gastrointestinal complaints. Table 1 lists the scenarios for CeD screening in individuals having fragility fractures or low BMD.

## 4. General Non-Pharmacological Management of Osteoporosis in Adult-Onset Celiac Disease

### 4.1. General Measures

Several interventions are necessary for all CeD patients to promote bone strength, including adequate vitamin D and calcium intake; cessation of tobacco use; avoidance of excessive alcohol consumption; regular weight-bearing and muscle-strengthening exercise; and identification of fall risk factors, such as impaired visual acuity and sedative medications [63].

Celiac patients with low BMD should be encouraged to increase physical activity and undertake lifestyle changes such as smoking cessation, as well as moderate coffee consumption (up to 3 cups/day) [64,65] and avoidance of excess alcohol. In coexistent type 1 diabetes mellitus, good glycemic control is needed. Regulation of thyroid function is essential.

Physical activity. Several studies [66,67] established the beneficial effect of physical activity in lowering the risk of developing osteoporosis [66,68]. From studies in elderly individuals, with or without osteoporosis but without CeD, we know that regular exercise, in particular resistance and high-impact activities, contributes to the development of high peak bone mass and may reduce the risk of falls in older subjects. Physical activity was clearly associated with a lower risk of humerus and femoral neck fractures [66]. High-impact activities and resistance training appear to be effective in reinforcing BMD at the hip and spine in premenopausal women, according to a meta-analysis [67].

For those who have already suffered osteoporotic vertebral fractures, there are specific exercise recommendations: aerobic physical training, postural corrections, resistance, balance training, and exercises for the trunk and lower extremity muscles. These exercises aim to improve and maintain the stability of the spine, optimize functional performance, and minimize the danger of falls and fractures [69].

Smoking cessation. Nicotine exerts a toxic effect on osteoblasts and is associated with a reduced vitamin D level in the blood; therefore, smokers have lower BMD and approximately a 55% higher risk of hip fracture than non-smokers [70].

Furthermore, a smoker heals poorly because of poor blood supply and, consequently, it takes longer to recover BMD after a fracture [71].

Avoidance of excess alcohol. Average or ordinary alcohol intake is not usually related to negative effects on BMD. On the other hand, consuming more than two drinks per day for women or three drinks per day for men may be harmful to bone health [72]. This could be related to the association between decreased absorption of calcium and a heightened risk for falls. Individuals who are at risk of heavy alcohol consumption and/or binge drinking should seek appropriate advice or treatment. Consumption of alcohol increases the risk of fracture non-union. It has been shown that alcohol deranges Wnt signaling, which is needed for repair of fractures [71,73]. In addition, alcohol abuse is of particular interest because nearly half of orthopedic trauma patients have elevated blood alcohol levels at the time of injury.

Hormonal deficiencies. This is usually an indication for referral to an endocrinologist. In cases of estrogen deficiency, estrogen therapy needs to be provided when there are no contraindications [74]. In men with hypogonadism, testosterone therapy may be considered to improve both hypogonadal symptoms and BMD [75,76].

### 4.2. Gluten-Free Diet

Undoubtedly, GFD is the most effective treatment for CeD and other gluten-related disorders. It means abstinence from wheat, barley, rye, green spelt, spelt, and all gluten-containing ready-made products. However, complete abstinence from gluten-containing products is somewhat difficult.

The excess risk of osteoporosis may be minimized through strict adherence to a GFD which is necessary for the healing of villous atrophy [77]. Studies have reported that adherence to GFD has a beneficial impact on maintaining bone density [26,78]. There is evidence to suggest that BMD increases during the one or two years up to 5 years of GFD, particularly in those younger than 40 years of age [26,27,77,79]. GFD benefits elderly patients with newly diagnosed CeD, with significant gains in BMD at the lumbar spine and femur in the first 12 months of treatment [78,80]. However, convincing evidence about achieving and maintaining normal bone mass is lacking. In children, a gluten-free diet positively influences bone metabolism because of a reversal of the inflammatory process [81].

In the high-risk group of patients with malnutrition, the recovery of BMD is delayed, while well-nourished patients may show a progression from osteoporosis to osteopenia [79,82,83,84].

Overall, studies have shown that a GFD is an effective therapy for the durable recovery of BMD. Additionally, fracture risk declined in a group of patients compliant with strict GFD at 5 years after diagnosis [77]. It seems that GFD had a positive effect on bone turnover markers compared to untreated subjects [85].

Adherence to GFD needs to be checked periodically. Clinical parameters, dietary reviews, use of special questionnaires, serology parameters, and even follow-up duodenal biopsy are needed [86]. All celiac-specific antibodies are dependent on exposure to gluten, and a decrease in titer from baseline values is anticipated within weeks to a few months of following a strict diet. A positive anti-tissue transglutaminase antibody after GFD indicates continued, albeit minimal contamination, gluten ingestion and is associated with continued enteropathy. Detecting gluten immunogenic peptides in stool or urine might provide evidence of continued gluten ingestion [87,88]. Hence, because of some shortcomings, there is as yet no consensus on using this test in clinical practice [5].

### 4.3. Vitamin D and Calcium

Adherence to strict GFD for at least 1–2 years results in the normalization of vitamin D and calcium levels. In this phase, supplementation is usually necessary. There is a subset of patients who need long-term supplementation, such as postmenopausal women, malnourished patients, and those with low BMD [89].

Vitamin D and calcium should be given to those patients who have a considerable risk of fractures due to bone fragility. The amount of calcium necessary for optimal bone health differs at different ages; it is higher in teenagers when there is a rapid growth rate of the skeleton and at an older age, when the ability to absorb calcium declines. There is substantial variability in recommended calcium requirements between countries and guidelines [90]. The Food and Agriculture Organization (FAO) Recommended Nutrient Intakes are 800–1000 mg of calcium per day in men and women over the age of 50 years [91]. The Institute of Medicine of the United States National Academy of Sciences recommends 1000 mg per day for women aged 19 to 50 and men aged 19 to 70, and 1200 mg per day for postmenopausal women and men aged 70 and up [92]. For celiac patients, the daily calcium intake needs to be higher than the recommended daily allowance. This may compensate for adult celiac patients’ reduced fractional calcium absorption [93]. Adequate amounts of calcium can be provided by ingestion of dairy products, some dark greens and vegetables, fruits, fish with bones, and fortified foods.

The factors that need to be taken into consideration before selecting a calcium supplement are the presence of impairment of gastric acid secretion, lactose intolerance; and the risk of kidney stone formation. There is evidence that calcium supplementation of more than 1200–1500 mg/day might increase the chance of kidney stones in some individuals at-risk.

Calcium citrate and calcium carbonate are the two anions in the available calcium supplements [94]. Absorption of the citrate anion is better than that of carbonate and is also not influenced by changes in gastric acidity, producing a larger rise in serum calcium and a decrease in parathormone [94].

Vitamin D dosage depends on skin type, age, season, and geography. The main dietary sources of vitamin D include fortified milk and cereals, fish (such as tuna, salmon, and mackerel), and oil from cod fish liver. Supplementation with either vitamin D_2_ (ergocalciferol) or vitamin D_3_ is usually effective, but cholecalciferol is preferable. Ergocalciferol is obtained from plant sources and therefore would be a good alternative for those on a vegetarian or vegan diet.

The Institute of Medicine (IOM) in the USA and the European Food Safety Authority (EFSA) have published detailed guides on vitamin D requirements [92,95]. In summary, the IOM’s and EFSA’s stated Estimated Average Requirement (EAR), Recommended Dietary Allowance (RDA), and Adequate Intake (AI). The IOM set the Dietary Reference Intakes (DRIs) for vitamin D for adults at 10 µg (EAR) and 15 µg (RDA). The EFSA Panel defined the adequate intake (AI) value at 15 µg.

The following need to be monitored at follow-up visits: serum calcium, renal function, serum alkaline phosphatase, 25(OH) vitamin D and parathyroid hormone [96].

### 4.4. Magnesium

Magnesium deficiency, when documented, needs to be corrected. However, data on the effectiveness of magnesium supplementation on bone metabolism are inconclusive [97].

## 5. Pharmacological Management of Osteoporosis in Adult-Onset Celiac Disease

### 5.1. Indications for Pharmacological Treatment

Osteoporosis in patients with CeD is a clear indication for pharmacological treatment. However, osteopenia in CeD in itself is not enough to institute pharmacological therapy, unless in high-risk patients, especially those with a severe course of the disease and worsening DXA bone mineral density values, and in patients with vertebral fractures. The discovery of one or more vertebral fractures with more than 25% vertebral height loss, especially in the lower range of osteopenia, T-score of −2.0 and lower, can lead to the initiation of anti-osteoporotic treatment [54].

### 5.2. Overview of Osteoporosis Medications

The currently approved pharmacological agents for the prevention and/or treatment of osteoporosis are bisphosphonates, denosumab, parathyroid hormone, an analog of parathyroid hormone-related peptide, calcitonin, estrogens, estrogen agonist/antagonist, tissue-selective estrogen complex, and the monoclonal antibody to sclerostin (romosozumab).

The clinical data available on the reduction in fracture risk of these drugs have been studied primarily in postmenopausal women. Data are limited regarding efficacy for secondary osteoporosis (such as celiac disease, Inflammatory Bowel Disease (IBD), diabetes, and therapy with glucocorticoids). There is also a scarcity of data on the treatment of men with osteoporosis. However, the results of clinical trials may be extrapolated to CeD until more specific data are available. Table 2 summarizes important data regarding these agents.

### 5.3. Medications: Selection, Regimen, Duration and Side Effects

The choice of a certain pharmacological agent needs to be based on factors such as osteoporosis severity, fracture history, hip fracture risk, comorbidities (e.g., peptic ulcer, gastroesophageal reflux disease, malabsorption syndrome, etc.), financial cost, and others [115]. Furthermore, preference for a specific oral, subcutaneous, or intravenous compound depends on many factors, such as a drug’s oral bioavailability, established effectiveness, and easiness of application with low frequency, such as in intravenous derivatives, such as pamidronate, ibandronate, or zoledronate, or subcutaneous denosumab. Particularly in the early years after the diagnosis of CeD, intravenous preparations may be preferable because of the anticipated decreased oral bioavailability due to villous atrophy.

The duration of bisphosphonate treatment in CeD is less well-delineated. It appears sensible to follow an approach akin to senile osteoporosis and to reevaluate treatment after a maximum of five years. Afterward, they decide to discontinue treatment except for those with continued high-risk or fragility bone fractures.

Treatment with any of the osteoporosis agents has its own limitations. Bisphosphonates’ use is limited to 3–5 years; denosumab can be prescribed for up to 10 years, but stopping the treatment might induce vertebral fractures, while parathormone analogs are limited to 2 years. Short-term use of any of these agents is not capable of preventing the large majority of fractures in the course of a patient’s lifetime [116]. Some residual reduction in fracture risk is sustained after withdrawal of teriparatide [117], but this effect fades and disappears over time. Sequential treatment is therefore suggested to maintain the gain of BMD achieved by therapy with anabolic agents. One regimen that is gaining acceptance is starting with an anabolic agent, followed by an antiresorptive medication [118,119,120].

In terms of side effects, osteonecrosis of the jaw (ONJ) and atypical femoral fracture are uncommon but serious complications associated with bisphosphonates [121]. The majority of ONJ cases occur in oncology patients, in whom more frequent and higher dosages are used. The risk of ONJ is estimated to be 1/100.000 patient-years in osteoporosis and up to 100/100.000 patient-years in oncology cases treated with bisphosphonates [122]. Dento-alveolar surgery, periodontal disease, and concomitant oral glucocorticoid use constitute risk factors for ONJ. The incidence of atypical femoral fracture is associated with the duration of bisphosphonate therapy [123]. It is necessary to evaluate patients for these adverse events at 3 and 5 years of treatment using zoledronic acid and alendronate, respectively. As a result, those who are at high risk at the time of evaluation (high fracture risk score or low BMD) require ongoing treatment as well as regular evaluation [124].

Regarding romosozumab, it is a new strong anabolic drug, that reduces within 1 year the fracture rate at both vertebral and nonvertebral sites, even when compared with alendronate [118,125] or denosumab [126]. However, there is some concern about a possible increase in cardiovascular events [114,127], and it is only indicated for severe postmenopausal osteoporosis [128], and not for men.

Table 3 provides an overview of the dosing schemes of important medications for osteoporosis in CeD patients.

## 6. Practical Management of Osteoporosis in Adult-Onset Celiac Disease

### 6.1. Premenopausal Celiac Disease Women with Osteoporosis

Fractures due to osteoporosis are considerably less frequent in premenopausal than in postmenopausal women [129]. However, fractures in premenopausal women may be a good indicator of underlying poor quality of bone health and may predict future fracture risk. There is no universal agreement on criteria to diagnose osteoporosis in premenopausal women [129,130]. Most guidelines recommend that the diagnosis can be made in women with clinically manifest hip or vertebral fragility fractures, or when fragility fractures in other skeletal sites are combined with low BMD. Furthermore, to classify BMD in these patients, there is no clear agreement on using a T-score or Z-score (in reference to age-matched controls) [130,131]. However, some experts advocate utilizing a T-score > –2.5 for making a diagnosis of osteoporosis in these women because this is a clear cut-off value familiar to a wide range of clinicians [74].

The general treatment measures mentioned above should be recommended. In those women who have recurrent fractures or when there is a worsening of osteoporosis, pharmacological intervention may be considered [74]. Before starting medications, future pregnancy plans should be discussed. Bisphosphonates affect the fetal skeleton adversely as they adhere to bones for a long time and can traverse the placenta [132]. If there are no plans to get pregnant, therapy may be commenced, but it should be stopped at least 6 months before attempting to become pregnant. Women receiving teriparatide should be warned to be very cautious and not to become pregnant.

As denosumab has a shorter half-life relative to bisphosphonates and lacks the property of skeletal accumulation, it may have advantages in premenopausal women, but its efficacy and safety have not yet been defined in these women [133].

### 6.2. Treating Male Celiac Patients with Osteoporosis

In general, osteoporosis in men is usually secondary and mostly linked with comorbidities. Therefore, detection of the cause of osteoporosis is crucial to instituting directed treatment. Further, men have an increased rate of complications related to osteoporosis and also high fracture-related mortality [134]. Interestingly, only a minority of male osteoporosis patients receive a well-timed diagnosis and fewer get appropriate therapy. Importantly, very few studies investigated the efficacy of anti-osteoporotic treatments in men [134,135].

It is recommended to test higher-risk men (aged ≥70 years and men at younger ages who have risk factors for secondary osteoporosis using DXA) [115]. Celiac disease is one of these factors.

In addition to the general measures mentioned above, pharmacological treatment is recommended for men who suffered a low-impact spine or hip fracture without major trauma, those having T-scores of ≤−2.5, and those patients who have low BMD and/or clinical risk factors that increase risk of fracture [115].

### 6.3. Monitoring and Follow-Up

Those patients who do not require pharmacological agents at the time of initial evaluation should be clinically followed and reevaluated when medically indicated.

International guidelines on monitoring response to osteoporosis treatment recommend that treatment should be monitored with serial DXA testing [136,137]. The recommended interval is every 2–3 years, as it takes longer for the BMD to change significantly. Renal function and serum levels of calcium, phosphate, alkaline phosphatase, vitamin D, and parathormone need to be carefully monitored.

Elevated serum levels of parathormone, osteocalcin, bone-specific alkaline phosphatase, and low 25-hydroxyvitamin D_3_ levels have been proposed as markers of ongoing bone disease in the follow-up of CeD patients [138].

However, guidelines suggested measuring bone turnover marker(s) (when available) 3–6 months after starting therapy to monitor changes in BMD because a change in these markers is faster than in DXA [139,140]. These markers are not widely available for daily practice.

In order to detect any new vertebral fractures, radiological examinations need to be repeated if there is a loss of height, development of new back pain, or a change in posture.

Regularly, the treating physician needs to assess compliance with the therapeutic management regimen no less than yearly.

During follow-up, continued loss of BMD at a high rate despite appropriate vitamin D and calcium supplementation necessitates reassessing GFD adherence, taking a follow-up duodenal biopsy, and revision of ancillary risk factors. In addition to consultation with a bone-specialist.

### 6.4. Consultation with Bone Specialist

A consultation for endocrinology, rheumatology, or geriatrics should be considered in the following scenarios [5,14]:

- Continuous unexplained bone loss;

- The presence of concomitant rheumatological disorders;

- The presence of hyperparathyroidism, hypogonadism, or other endocrine disorders;

- If the treating gastroenterologist does not feel confident enough in treating bone disease adequately.

## 7. Conclusions

Patients with celiac disease are at high risk for osteoporosis and fractures due to nutritional malabsorption and deficiencies, inflammation-induced high bone resorption, and decreased overall physical health and mobility. Awareness of the celiac disease is needed, as it is often silent or mono-symptomatic in adults.

Optimal treatment of celiac disease and an adequate supply of calcium and vitamin D are the cornerstones of fracture risk reduction in patients with celiac disease. A targeted case-finding approach for celiac disease is recommended in adults with low BMD or fragility fractures.

In patients with a high risk of fracture, antiresorptive drugs need to be prescribed to counteract the elevated bone resorption. Due to the malabsorption in patients with celiac disease and because oral bisphosphonates such as alendronate and risedronate have only absorption of 1–3% in postmenopausal osteoporotic women, parenteral treatment options, such as zoledronic acid or denosumab, are preferred above oral medications. This may be particularly crucial in the early years of treatment. Research directed at this practical management issue in celiac disease patients is needed.

Anti-osteoporosis medications may also be indicated in patients with severe osteopenia who have an increased risk for fractures or in the case of severe celiac disease diarrhea/steatorrhea or refractory celiac disease.

We confirm that the literature directed at the prevention and treatment of decreased BMD and fractures in relation to celiac disease is scarce. Therefore, directed research to provide evidence-based recommendations specific to abnormal bone mineral density in celiac disease is mandatory.

## Figures and Tables

**Table 1:** The scenarios for CeD screening in individuals having fragility fracture or low BMD.

1	Vitamin D deficiency, elevated parathyroid hormone or low urinary calcium level despite sufficient vitamin D and calcium intake *.
2	When there is an inadequate response to therapy with oral bisphosphonates
3	The presence of medical conditions having a clear risk of possible celiac disease development: Autoimmune disorders of the thyroid gland and liver diseases Dermatitis herpetiformis ^†^ Type 1 diabetes mellitus Down syndrome Persistent unexplained steatorrhea after upper gastrointestinal surgeries
4	The presence of any symptoms suggestive of celiac disease Gastrointestinal symptoms Chronic non-bloody diarrhea ^†^ Signs and laboratory findings of malabsorption ^†^ An otherwise unexplainable weight loss ^†^ Iron deficiency with or without anemia without evident blood loss ^†^

* Adequate daily vitamin D and calcium intake (see text for recommended daily allowance). ^†^ Patients with these conditions, symptoms, or signs should have histological examination of duodenal biopsy regardless of the results of celiac disease serologic testing. CeD, celiac disease; BMD, bone mineral density.

**Table 2:** Summarizes important data over pharmacological agents used for osteoporosis.

Category	Summary	
**1. Antiresorptive Agents**		
1a. Bisphosphonates		
Examples: Alendronate and risedronate (orally), ibandronate (orally or intravenously), Pamidronate (intravenously) and zoledronic acid (intravenously). Usually, the 1st line in management	Mechanism of action	The antiresorptive effect of bisphosphonates is derived from the affinity for hydroxyapatite and their inhibitory capability to the osteoclast enzyme farnesyl pyrophosphate synthase [98]. They promote apoptosis of osteoclasts resulting in the inhibition of bone resorption and an increase in BMD.
	Evidence of efficacy	In postmenopausal osteoporosis, these agents improve BMD at the spine (risk reduction 4–9%) and hip (2–6%) after 3 years [99] and decreased fracture risk in comparison with placebo [100]. They also improve BMD in cohorts of Crohn’s patients [101]. A critical issue is that only 1–3% of bisphosphonates are absorbed. In celiac disease, this might be lower, indicating that oral bisphosphonates might be ineffective in active celiac disease versus those in remission. Bone markers, such as CTX (bone resorption) and P1NP (bone formation) should be decreased by 30% or more after 3–6 months of therapy, a high level may indicate poor absorption. In those with poor response, switching to intravenous bisphosphonate is indicated.
	Important side effects/drawbacks	Adverse events of bisphosphonates are uncommon. Oral administration of bisphosphonates may be associated with dysphagia, abdominal pain, nausea, constipation or diarrhea, acid regurgitation, taste distortion, gastritis and esophageal ulcers. Hypocalcemia is reported particularly after starting potent intravenous bisphosphonates such as zoledronic acid. It is strongly recommended to commence the substitution of calcium and vitamin D 2–4 weeks before bisphosphonates to ameliorate the risk of tetany [102]. The incidence of osteonecrosis of the jaw (ONJ) is small in patients using bisphosphonates for osteoporosis prevention or treatment, ranging from less than 1–28 cases per 100,000 person-years of treatment [103]. In cancer patients, a study showed that ONJ developed in 1.4% of those who were treated initially with zoledronic acid, over the course of 5 years [104].
1b. Monoclonal antibodies		
Denosumab 1st or 2nd line in management	Mechanism of action	It is the most powerful antiresorptive agent [105], a monoclonal antibody to the RANKL; which is a key regulator of bone resorption
	Evidence of efficacy	In postmenopausal women, subcutaneous denosumab 6-monthly improved the BMD in both the spine (9%) and the hip (6%) [106]. It reduced the fracture risk: hip (40%), and nonvertebral (20%). In contrast to bisphosphonates, long-term denosumab results in continued improvement in BMD [107]. Further, switching to denosumab in those who had long-term bisphosphonates induced greater BMD gains over 12 months of treatment [108]. Therefore, this approach may be preferable when a response to intravenous bisphosphonates is inadequate.
	Important side effects/drawbacks	The effect of denosumab is reversible, thus a loss in BMD may happen after treatment cessation [109]. Therefore, it is advocated to continue with an antiresorptive agent after stopping denosumab [110].
**2. Anabolic agents**		
2a. Parathyroid hormone 2nd or 3rd line in management	Mechanism of action	The recombinant parathyroid hormone fragment (1–34 or teriparatide) results in increased bone formation when administered intermittently [111].
	Evidence of efficacy	Used to treat corticosteroid-related bone loss and also in postmenopausal osteoporosis with beneficial effects on BMD and reduction in vertebral and nonvertebral fractures.
	Important side effects/drawbacks	Many guidelines restrict its use to two years in those patients at high risk of/or documented vertebral fractures [96] because of an increased risk of osteosarcoma found in studies in rodents, but this has not been seen in human studies. Therefore, the new teriparatide label [112] states that use for more than 2 years may be allowed in patients having a high risk for fracture. It needs to be avoided when there is a heightened risk for osteosarcoma. Its effect is reversible, thus if discontinued a decline in BMD may follow.
2b. Romosozumab2nd or 3rd line in management. The main indication is postmenopausal osteoporosis.	Mechanism of action	A humanized monoclonal antibody to sclerostin (a glycoprotein blocks canonical Wnt signaling bone formation pathway). It improves bone strength by increasing bone formation and suppressing bone resorption
	Evidence of efficacy	Monthly subcutaneous administration (maximal 12 months) decreases the occurrence of vertebral fractures in postmenopausal osteoporosis [113].
	Important side effects/drawbacks	There is concern about a possible increase in cardiovascular events [114]. It is mainly indicated for severe postmenopausal osteoporosis.

Abbreviations: BMD = bone mineral density; ONJ = osteonecrosis of the jaw; RANKL = receptor activator of nuclear factor kappa-B ligand; CTX = serum C-telopeptide of type I collagen; P1NP = procollagen 1 N–terminal propeptide.

**Table 3:** Overview of dosing schemes of medications for osteoporosis in celiac disease patients.

Medication	Dosing Scheme; Route of Administration	Duration of Therapy
Bisphosphonates		5 years
Alendronate	70 mg per week; orally	
Ibandronate	150 mg, monthly. Orally. Or 3 mg every 3 months; intravenously	
Pamidronate	Different schemes, intravenously. e.g., 90 mg every 4 weeks	
Risedronate	35 mg weekly. Orally	
Zoledronic acid	5 mg, once yearly; intravenously	
Denosumab	60 mg twice yearly; subcutaneously	5 years, followed by another antiresorptive therapy
Teriparatide	20 micrograms daily; subcutaneously	2 years; then 1st or 2nd line agent (see Table 2)
Romosozumab	210 mg (administered as two subcutaneous injections of 105 mg each) once a month	One year. Thereafter start antiresorptive therapy with bisphosphonate

Notes: Sequential therapy [115,118,119,120]: one regimen that is getting acceptance is starting with an anabolic agent, to be followed by an antiresorptive medication. This is because short-term use of any of these agents is not capable of preventing the large majority of fractures in the course of a patient’s lifetime.

## Data Availability

Not applicable.
